# Untapped microbial factories for next-generation therapeutics: Endophytic fungi of medicinal plants

**DOI:** 10.1186/s12934-026-03036-4

**Published:** 2026-06-04

**Authors:** Abdullah M. Nagib, Mahmoud H. Sultan, Hussein H. El-sheikh, Amr H. Hashem

**Affiliations:** 1 Faculty of Science, Al-Azhar University, Cairo, 11884 Egypt; 2 Botany and Microbiology Department, Faculty of Science, Al-Azhar University, Cairo, 11884 Egypt

**Keywords:** Endophytic fungi, Bioactive compounds, Biomedical applications, Multi-omics

## Abstract

Endophytic fungi residing within medicinal plants represent a rich and sustainable source of bioactive secondary metabolites with diverse pharmacological applications. These symbiotic microorganisms establish mutualistic relationships with their host plants, contributing to enhanced stress tolerance, growth promotion, and defense against pathogens. In recent years, endophytic fungi have gained considerable attention as alternative biofactories for the production of valuable compounds such as alkaloids, terpenoids, phenolics, lignans, and polysaccharides. These metabolites exhibit a wide range of biological activities, including antimicrobial, antiviral, anticancer, antioxidant, anti-inflammatory, and antidiabetic effects. Advances in cultivation strategies, such as OSMAC, co-culture, and epigenetic modification, have significantly improved metabolite yield and diversity. Moreover, the integration of genomics, transcriptomics, and metabolomics has revolutionized the understanding of biosynthetic gene clusters and metabolic pathways, enabling the discovery of novel compounds and optimization of production processes. Despite these advances, challenges such as low yield, silent gene clusters, and difficulties in large-scale production remain significant barriers. Nevertheless, continued progress in multi-omics technologies, synthetic biology, and biotechnological tools holds great promise for unlocking the full potential of endophytic fungi. Herein, endophytic fungi represent a powerful and eco-friendly platform for the development of new therapeutic agents and sustainable pharmaceutical applications.

## Introduction

Endophytic fungi are microbes that asymptomatically invade the intercellular and, more rarely, the intracellular tissues of a healthy plant at some time during its life cycle. Compared with pathogens, these fungi live in mutualistic, commensal, or latent forms within their host and frequently improve the health of the plant while deriving their nutrients from tissues and taking advantage of its habitat. They are widely distributed in almost all plant species from different habitats, such as medicinal plants, crops, and forest flora [1–3]. Endophytic fungi and their host plants have co-evolved over billions of years, resulting in a symbiotic relationship that offers the host several ecological and physiological benefits. These fungi assist plants to withstand different abiotic challenges, including dry spells, salinity, and temperature oscillations, by fabricating shielding metabolites [4]. They also promote disease resistance by the production of antimicrobial compounds that protect the plant from pathogens, pests, and herbivores [5]. Moreover, some endophytes enhance plant physiology by producing key phytohormones, including gibberellins, indole-3-acetic acid (IAA), and cytokinins, which collectively promote plant growth and improve nutrient uptake, particularly under nutrient-limited conditions [4, 6, 7]. In addition to these functions, endophytes are known to associate with plant metabolic pathways either positively or negatively, affecting the synthesis of such secondary metabolite compounds that increase stress tolerance of plants and have a potential advantage for human and veterinary medicine [8].

Medicinal plants are known for the abundance of bioactive phytochemicals, i.e., alkaloids, flavonoids, terpenoids, and phenolic compounds involved in their known antioxidant, antimicrobial, anti-inflammatory, and anticancer activities [9–11]. However, the production of these substances is usually obtained through massive collection, thus constituting a threat to the preservation of plant species. In this context, endophytic fungi have the potential to serve as an alternative source due to their diverse biosynthetic machinery. A few endophytes are able to imitate host plant metabolites by producing compounds similar to or identical to the ones produced by medicinal plants [2, 12]. Moreover, they are reputed to form new chemical analogues that increase or supplement the therapeutic capabilities of plant metabolites [13]. Besides their metabolic flexibility, these fungi can be produced and genetically modified, which facilitates the production of high quantities of valuable bioactive compounds in a sustainable manner, avoiding pressure on natural plant resources [14, 15].

Medicinal plants provide a chemically rich environment that supports diverse endophytic fungi, which contribute significantly to the production of valuable secondary metabolites. These fungi are producers of valuable medicinal drugs such as paclitaxel (Taxol) from *Taxomyces andreanae*, a widely used tumor growth inhibitor [15], and have also demonstrated the ability to produce antimicrobial agents active against antibiotic-resistant pathogens [16]. The volatile organic compounds (VOCs) produced by endophytic fungi have been found to be antimicrobial and growth-promoting, which can improve the plant tolerance under environmental stress, and have potential applications in agriculture and the medicinal industry [17]. Fungi reduce biotic stress and enhance nutrient metabolism, thereby contributing to the quality of plant secondary metabolites [4]. Certain classes of secondary metabolites, including flavonoids, glycosides, and phenolic compounds, are predominantly produced by endophytic fungi inhabiting aboveground plant tissues, rather than being synthesized by the host plant, demonstrating their contribution to chemical peculiarities in medicinal plants [12, 18]. They are also producers of antimicrobial and antiviral metabolites, such as alkaloids, peptides, and polyketides that have broad-spectrum antibacterial activity against antibiotic-resistant bacteria and new viral infections [5]. Moreover, the endophytic fungal metabolites have been shown to have antioxidative efficacy and protect cells from oxidative stress and some chronic diseases, including neurodegenerative and cardiovascular diseases [19, 20]. Aside from human health, endophytic fungi also play a role in agriculture and plant protection through enhancing crop immunity. They apply biocontrol mechanisms that help to prevent pests, and fertilization, sustain soil fertility, as well as the cultivation-promoting action of plants through the production of various bioactive compounds [4]. Endophytes represent a sustainable and prolific reservoir of valuable metabolites for use in biological, biotechnological, and agricultural applications, without placing excessive pressure on the overexploitation of medicinal plants. Their metabolic diversity and potential biomedical applications should continue to be explored for the development of new therapies, particularly in response to pressing global health challenges [15, 21–27].

The novelty of this review lies in its integrative and application-oriented approach that bridges recent advances in multi-omics technologies with practical strategies for strain improvement and scalable production of bioactive metabolites from endophytic fungi. Unlike previous reviews that primarily summarize metabolite diversity and biological activities, this work emphasizes the linkage between genomics, transcriptomics, and metabolomics with cultivation-based approaches such as OSMAC, co-culture, and epigenetic modulation to activate silent biosynthetic gene clusters and enhance metabolite yield. Furthermore, it highlights translational aspects by addressing current bottlenecks such as low productivity and industrial scalability and proposing solutions that connect laboratory discoveries with field and bioprocess applications, thereby offering a more comprehensive framework for developing endophytic fungi as sustainable sources of therapeutic compounds.

## Diversity of endophytic fungi in medicinal plants

Diversity and community composition of endophyte microbiomes associated with medicinal plants are reversed by a recurring set of fungal phyla and genera, but are strongly influenced by host, organ, habitat, and season. Among most medicinal plants, the endophyte community usually consists of members of the phylum Ascomycota that largely outnumber with Basidiomycota [28]. Generally, numbers range from 3 to 9 phyla, 20–60 + orders, and 100–200 + genera per host [29]. Certain genera, including *Aspergillus*,* Penicillium*,* Fusarium*,* Trichoderma*,* Colletotrichum*, and *Nigrospora*, are frequent fungal occurrences for several medicinal plants in numerous regions, which was also the case with *Phyllosticta*, *Diaporthe*, and *Neopestalotiopsis* on medicinal plant hosts [2, 30]. Specialized groups, particularly Dark septate endophytes (DSE) such as *Phialocephala* and *Cadophora*, can predominate in roots from extreme or alpine environments and potentially contribute to stress tolerance [28, 29]. New genera and many new species are still being described from the Medicinal ferns and herbs, highlighting the vast and still underexplored fungal diversity associated with medicinal plants [31].

### Diversity drivers and host or tissue specificity

Host identity and tissue niche are dominant factors in determining diversity, with roots exhibiting the highest richness in some systems, while leaves and stems dominate in others [32]. Environment or season (altitude, habitat type, summer/winter, or potted/field) greatly reorganizes communities [28, 29]. Every medicinal plant is expected to have some core taxa that could be involved in stress tolerance, metabolite production, and pathogen defense [28]. Numerous medicinal plants harbor diverse endophytic fungi, predominantly Ascomycetes, which can mimic and produce novel bioactive compounds comparable to plant metabolites. Many endophytic genera are shared between medicinal plants of different hosts and regions, with some occurring frequently (Table 1) such as *Alternaria*,* Cladosporium*,* Diaporthe/Phomopsis* and *Daldinia*, which have often been reported from the medicinally important plants, and they usually produce terpenoids, alkaloids, quinones, and phenolics with antimicrobial and anti-cancer properties, or antioxidant activities [2, 14, 30, 33].

**Table 1:** Documented medicinal plants and associated endophytic fungal taxa

Medicinal plant	Dominant endophyte	Importance	Refs.
*Taxus brevifolia*	*Taxomyces andreanae*	Produces Taxol, same as the host	[14]
*Azadirachta indica*	*Eupenicillium parvum*	Produces azadirachtin A and B, insecticidal terpenoids	[14]
*Catharanthus spp.*	*Fusarium oxysporum*	Produces vincristine‑like alkaloids	[34]
*Cinchona spp.*	*Phomopsis sp.*	Produces quinine‑related compounds	[34]
*Anisomeles indica*	*Colletotrichum yulongense*, *C. cobbittiense*, *C. alienum*, *Fusarium equiseti*	Plant-growth promotion, rich in alkaloids, phenolics, and flavonoids	[33]
*Oroxylum indicum*	*Colletotrichum gloeosporioides*, *Daldinia eschscholtzii*,* Ectophoma multirostrata*	C. gloeosporioides extract shows strong antioxidant & cytotoxic activity	[35]
*Dillenia indica*	*Daldinia eschscholtzii*,* Colletotrichum gloeosporioides*,* Cladosporium cladosporioides*	Seasonal/tissue‑specific communities, strong enzyme production	[36]
*Ephedra pachyclada*	*Penicillium*, *Talaromyces*, *Aspergillus spp.*	Strong plant‑growth promotion, IAA, phosphate solubilization	[37]

### Ecological roles

Endophytic fungi of medicinal plants are diverse and composed of host- and habitat-specific communities that benefit the hosts by increasing host fitness, stress tolerance, defense responses, and production of secondary metabolites, and participate in ecosystem processes. Endophytic fungi play a crucial role in enhancing host plant stress tolerance and facilitating habitat adaptation. DSE are the most prevalent root endophytes in Alpine medicinal plants and are linked to cold and drought stress tolerance (Table 2); *Cadophora*,* Leptosphaeria*, and *Tetracladium* type taxa facilitate persistence of these plants in high elevation, low temperature habitats [29]. Medicinal plants adapted to cold desert environments, such as *Arnebia euchroma*, harbor endophytic communities with diverse plant growth–promoting traits that facilitate their survival under nutrient-limited and arid conditions [38]. Among species and sites, functionally redundant endophytes provide similar stress-ameliorating services even as species composition changes, buffering plant performance against climate change [28, 29]. Beyond stress mitigation, Many endophytes act as biofertilizers by improving nutrient availability (e.g., phosphorus solubilization and nitrogen facilitation), producing phytohormones such as auxins and gibberellins, and promoting root system development, leading to increased biomass and enhanced yields in medicinal plants and model crops [2, 38]. Functional guild analysis reveals the presence of tons of symbiotrophs, including mycorrhiza-like and root-associated biotrophs, in cultivable medicinal plants, suggesting participation in nutrient exchange and root function [39]. Additionally, endophytic fungi contribute to plant defense, community buffering, and ecosystem cycling. Endophytes inhibit pathogens and herbivores through antibiotics, lytic enzymes, siderophore production, inducible defenses, and competition, acting as endogenous biocontrol agents [2, 39]. Pathotroph–saprotroph endophytes may commence decomposition of senescent tissues, recycling nutrients whilst living within the plant, connecting endophytism with litter decay and nutrient turnover [28, 39]. Moreover, these fungi can regulate the biosynthesis of secondary metabolites in medicinal plants, thereby affecting their chemical composition and therapeutic quality. Many endophytes also play a role in or mimic host secondary metabolism, contributing to increased deposition of phenolics and other bioactive compounds in medicinal tissues, and even producing their own antimicrobial, antioxidant, antitumor, and insecticidal metabolites [2].

**Table 2:** Ecological roles of endophytic fungi associated with medicinal plants

Example	Role	Main effect on host/ecosystem	Refs.
Alpine and cold-desert medicinal plants with DSE-rich communities	Stress tolerance	Cold, drought, salinity, and metal resistance	[38]
Ephedra pachyclada, Bletilla striata, Arnebia euchroma endophytes	Growth promotion	Biomass, root growth, nutrient use	[40]
Antagonistic/pathotroph symbiotroph endophytes in multiple medicinal hosts	Defense/Biocontrol	Disease and pest suppression	[41]
Endophyte-enhanced phenolics, diverse terpenoids, and other SMs	Metabolite modulation	Higher or novel medicinal compounds	[42]

### Environmental and physiological factors affecting diversity

The endophytic diversity of fungi in medicinal plants is severely influenced by both external environmental factors (climate, soil, altitude, geography) and internal plant factors (organ, age, growth stage, chemistry). These factors act as filters that decide which fungi can colonize, persist, and dominate within the host tissues. Regarding environmental determinants, geography and habitat play crucial roles, as site and production area significantly influence the richness and OTU composition of endophytic communities in medicinal plants such as *Cissampelos pareira*,* Erigeron breviscapus*,* Rheum palmatum*,* Arnebia euchroma*,* Gentiana spp.*,* and Ardisia crenata* [39, 43]. These factors collectively shape the diversity and structure of fungal endophyte communities. In situ diversity of arbuscular mycorrhizal (AM) fungi in roots and rhizosphere soils is strongly influenced by both soil properties, such as total phosphorus and nitrogen availability, and geography factors like latitude and altitude, but comparatively more so for the former factors than for geographic distance alone [44]. Soil chemistry and plant taxonomic richness affect the extent to which host plants structure their root-associated fungal communities, with higher plant species diversity supporting increased microbial diversity in roots [45].

Climate conditions further modulate community structure, where temperature and humidity, but especially precipitation, such as rainy versus dry or winter seasons, consistently drive changes in alpha and beta diversity, with richness often increasing during warm, moist, or monsoon periods [39]. Temperature is associated with endophyte species richness and diversity, for example, in *Pinus Armandi*, where climate change leads to differential genomic vulnerability of endophytes by region [46]. Geographic factors account for generally greater variation in endophytic communities than climate alone. However, climatic factors such as drought and temperature fluctuations directly influence both fungal community assembly and host plant growth, while interactions may be further enhanced through inoculation with microbiomes derived from native plants [47, 48]. Additionally, seasonal changes in solar radiation, humidity, and temperature modulate endophytic community structure and function, affecting colonization timing and metabolic activities [49]. Along elevational gradients, diversity often shifts in a non-linear manner due to the combined effects of temperature, moisture, and surrounding plant communities; aerial tissues such as leaves and stems tend to respond more strongly to altitude compared with roots, which may exhibit distinct or attenuated patterns [28, 29]. In addition, soil properties and rhizosphere characteristics, including pH, nutrients, and rhizosphere community, are associated with the endophyte structure, diversity in alpine roots and *Ardisia crenata*, which affect the functional guilds and metabolite accumulation [29]. Soil characteristics underpin the assembly of endophytic fungi. Soil pH, organic matter, and macronutrients (particularly nitrogen, phosphorus, and potassium) are strongly correlated with the structure of fungal communities. Ren et al. [50] reported that, for example, found that geographic variation in soil fertility was directly related to significant differences in both rhizospheric and endophytic root fungal diversity in *Scrophularia ningpoensis*, with higher levels of diversity associated with relatively balanced nutrient profiles of the soil. In a similar vein, a study focusing on the orchid *Bletilla sinensis* along an altitudinal gradient found that soil characteristics as well as environmental variables like temperature and moisture played important roles in defining endophytic fungal diversity; furthermore, specific fungal taxa displayed significant preferences for certain soil features [51]. Rhizosphere properties constitute the dynamic interface with the most active exchange of signals between plant and microbes, serving as a secondary yet vigorous filter to narrow down endophyte candidates from the external soil microbiome. The composition and quantity of root exudates comprising sugars, amino acids, organic acids, flavonoids, and strigolactones are pivotal in this process. These exudates serve as carbon sources and chemoattractants that promote the selection of beneficial fungi while repressing antagonistic taxa by antimicrobial compounds. In a study with the grass *Melica transsilvanica*, it was found that *epichloë guerinii*, as an above-ground endophyte, altered the host’s root exudation profile by facilitating the secretion of organic acids, amino acids, and sugar alcohols, reconfiguring the rhizosphere microbiome to help bacteria harboring beneficial functional traits [52]. Beyond environmental influences, host-related physiological factors exert equally significant effects. The specific plant organ represents a strong, consistent driver; the roots are often associated with the highest richness due to their proximity to soil and exudates, whereas leaves and stems tend to support distinct and more evenly distributed microbial assemblages [28, 29, 39, 43]. Plant organ types are one of the main factors determining endophytic fungal richness, diversity, and community structure in medicinal plants. Roots often have higher fungal richness and diversity than stems and leaves [53]. For example, in *Sophora alopecuroides*, roots had the greatest fungal diversity. Similarly, in olive trees, inflorescences had richer endophyte communities than fruits [54]. These endophytes preferentially colonize a particular growth stage in a host plant, where their physiological state and immune responses could differ, thus tailoring the community structure of invading mycobiota. However, adaptive mechanisms and tolerance to environmental factors differ between growth stages, which may impact the composition of the fungal community within the plant [55]. Moreover, microbial communities associated with roots are also affected by plant provenance and environmental conditions, indicating that intrinsic factors, such as age and development, and extrinsic factors contribute to endophytic diversity [56]. The identity of host species and genotypes is also related to selective filtering, as closely related *Gentiana* spp. growing in the same region can result in different levels of endophytic communities and diversity, reflecting genetic and physiological traits [57]. Finally, the secondary metabolite profiles of a plant might influence microbial equilibrium; antibacterial or specialized compounds, such as those found in *Serjania erecta*, may alter dominance between bacterial and fungal or yeast communities, where endophyte diversity has often been reported to be positively correlated with major biochemical substances of the host, including anthraquinones, iridoids, shikonin, and dendrobine [58].

## Bioactive compounds produced by endophytic fungi

**Fig. 1:**
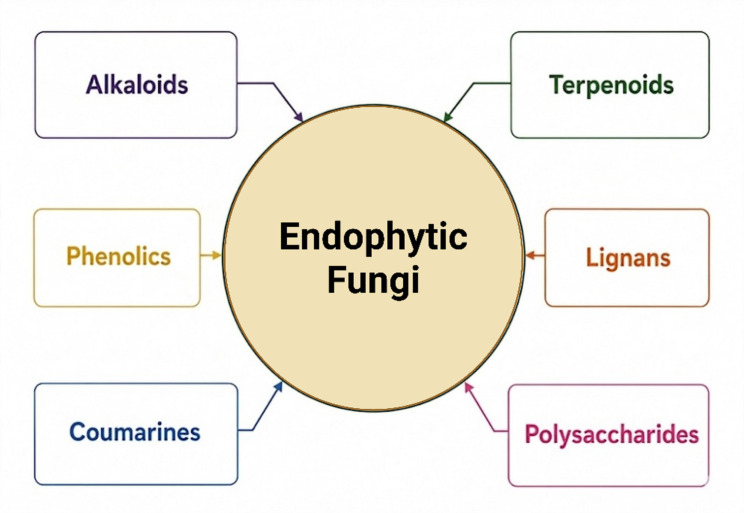
Bioactive metabolites of endophytic fungi from endophytic fungi. Created in BioRender. Hosny, A. (2026) https://BioRender.com/22xvb0w

**Fig. 2:**
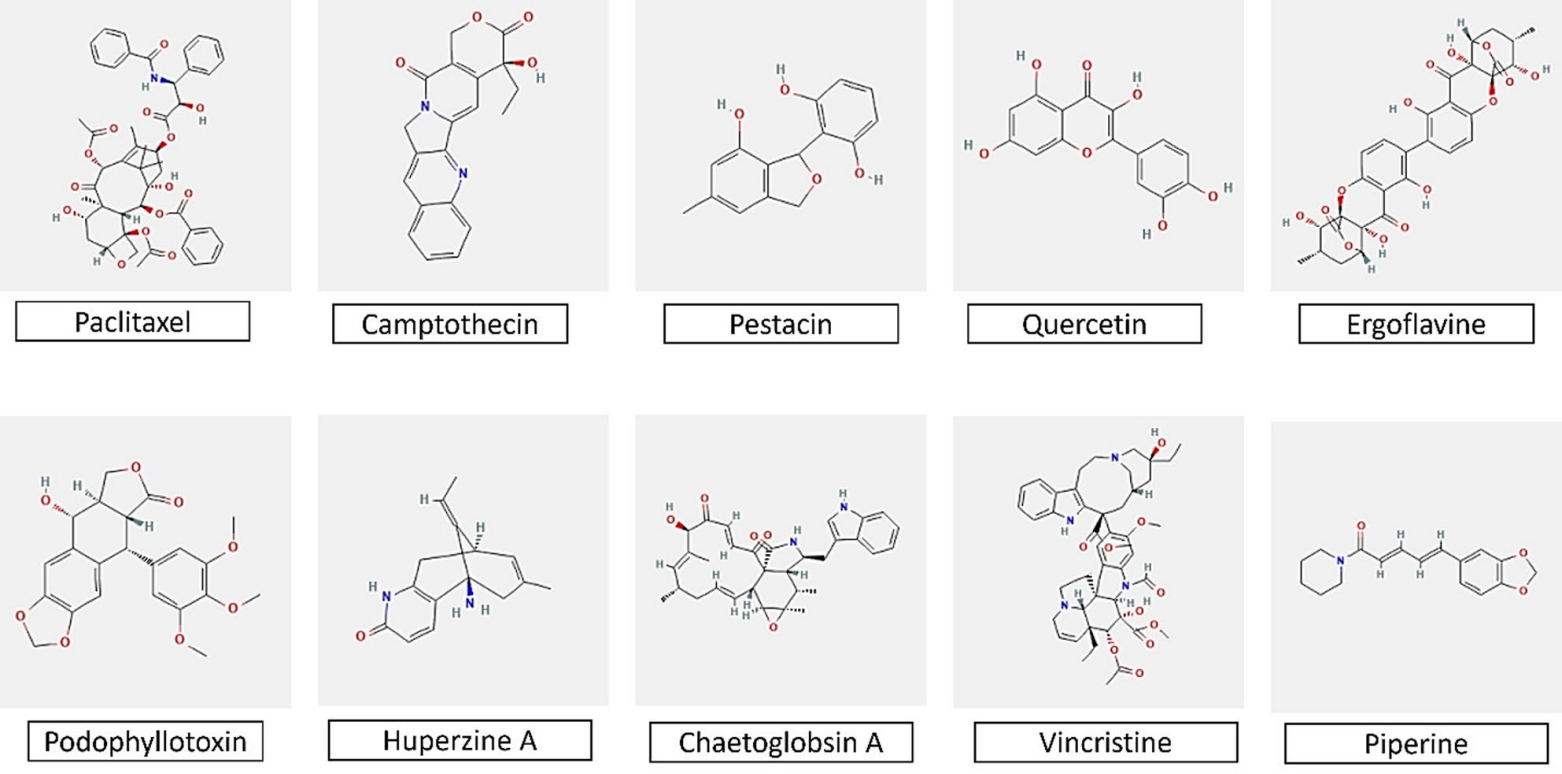
Some of common bioactive compounds from endophytic fungi

Endophytic fungi colonize the healthy internal tissues of plants and function as “chemical factories” to produce various secondary metabolites with potent pharmacological activity, like those made by plants. The primary bioactive classifications consist of alkaloids, terpenoids, phenolics, lignans and coumarins, polysaccharides, and various other metabolites (Fig. 1) [59, 60]. Recent research efforts have increasingly focused on endophytic fungi as promising sources of novel bioactive compounds with potential applications in multiple sectors [61]. Endophytic fungi have been considered as efficient biological factories due to their adaptability, ease of cultivation, and rich metabolic potential [62]. The discovery of the ability of the endophytic fungus *Taxomyces andreanae* to produce paclitaxel marked a breakthrough, underscoring the potential of endophytes as alternative sources of plant-derived metabolites [63]. Most of the reviews describe alkaloids as a primary metabolite class produced by endophytic fungi [60]. They are found in numerous hosts, including (medicinal plants, crop plants, trees), and genera (*Aspergillus*,* Penicillium*,* Fusarium*,* Colletotrichum*,* or Trichoderma*) [13, 64]. Often, terpenoids are the major new structures reported (35% of 220 new compounds in a decade) [60], such as monoterpenoids, sesquiterpenoids, diterpenoids, or triterpenoids with anticancer, anti-inflammatory, antibacterial, antiviral, and antimalarial activity [42, 64]. Phenolics (simple phenols, polyphenols, flavonoids) and coumarins and lignans exhibit potent antioxidant, antimicrobial, and cytotoxic activity. Polysaccharides and Other Metabolites: Fungal endophytes are recognized as prolific producers of diverse metabolites, such as polyketides, cyclic peptides, saponins, quinones, xanthones, macrolides, fatty acids, carotenoids, sugars, and polysaccharides [59, 65]. Figure 2 illustrates some of common bioactive compounds from endophytic fungi such as paclitaxel [66], camptothecin [67], pestacin [68], quercetin [69], ergoflavine [70], podophyllotoxin [71], huperzine A [72], chaetoglobsin A [73], vincristine [74], piperine [75].

**Fig. 3:**
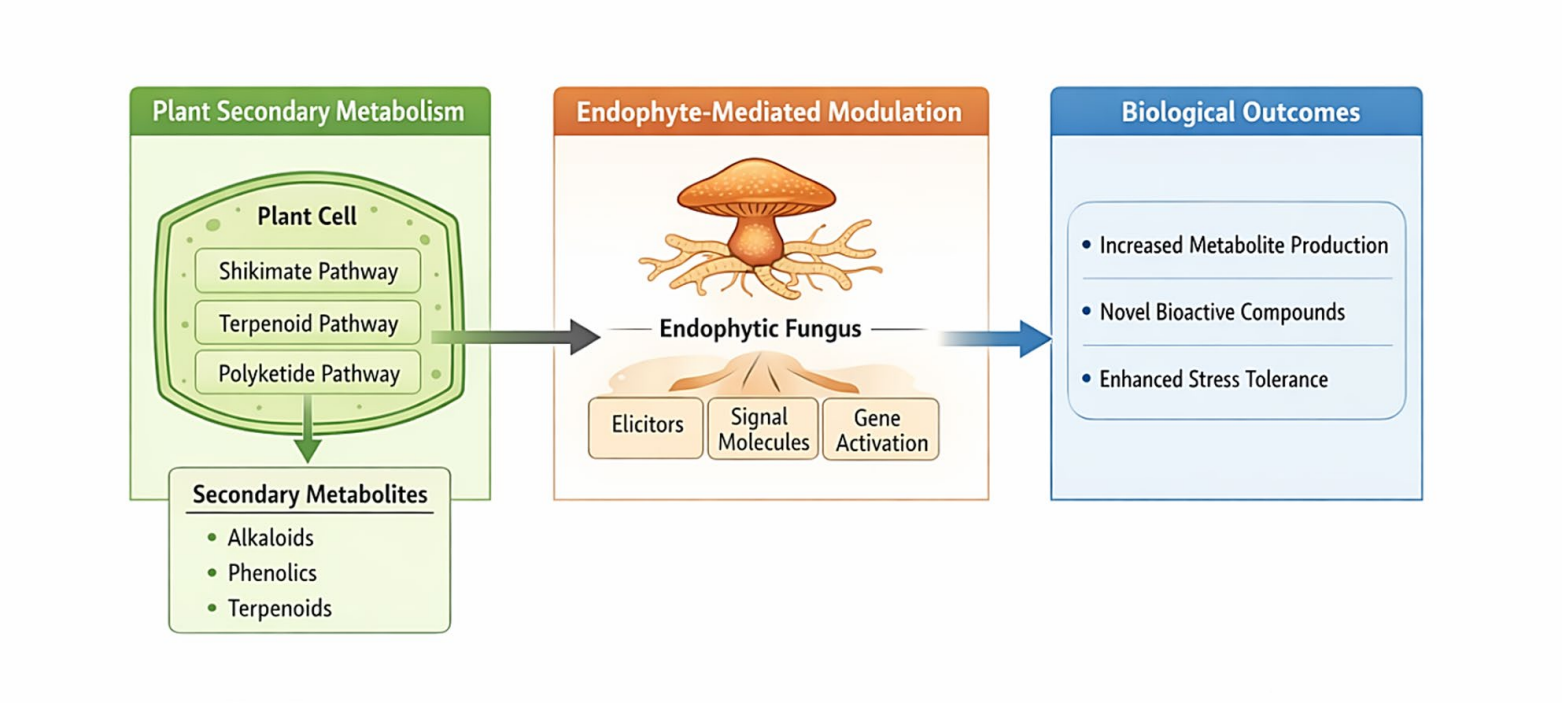
Plant secondary metabolite biosynthesis and its modulation by endophytic fungi

Plant secondary metabolites (PSMs), including phenolics, terpenoids, and alkaloids, are very important for plant defence and stress tolerance [76, 77]. Endophytes can change this metabolism by enhancing or redirecting PSM biosynthesis and, in some cases, contributing their own metabolites (Fig. 3) [40, 78]. PSMs are derived from biosynthetic pathways, including the shikimate/phenylpropanoid, terpenoid, and alkaloid pathways, and they are regulated by transcription factors (TFs) such as MYB, bHLH, WRKY, AP2/ERF, bZIP, and NAC [77, 79]. Biotic and abiotic stresses, such as salinity, temperature, drought, light, and herbivory, affect PSM accumulation through hormone signaling pathways and stress-inducible TFs [80, 81]. Endophytic fungi can increase the activity of biosynthetic enzymes like stilbene synthase and whole metabolic pathways, often through host TFs and stress-related signaling mechanisms [33, 78]. Additionally, fungal cells, extracts, or polysaccharides act as elicitors, enhancing antioxidant defenses and triggering the accumulation of defense-related PSMs [82]. Many fungal BGCs remain silent under standard conditions; however, approaches such as co-culture or the use of epigenetic modifiers can activate the production of novel fungal metabolites that may complement or mimic plant-derived PSMs [83]. Strains that enhance one class of metabolites may suppress others, reflecting a trade-off in resource allocation between growth and defense [84].

### Alkaloids

Alkaloids are low molecular weight organic molecules containing nitrogen atoms that derive from the amino acids and have a cyclic structure [65]. In endophytic Aspergillus, 263 alkaloids have been reported, such as cytochalasans, diketopiperazine, quinazoline, quinoline, indole, and pyrrolidine alkaloids [85]. Alkaloids constitute one of the major classes of secondary metabolites in endophytes, accounting for approximately 25–30% of newly identified structures [60]. Reported activities ranged from antibacterial, antifungal, antiviral, cytotoxic, and anticancer, anti-inflammatory, to antimalarial and cytostatic activities, in addition to enzyme inhibition (α-glucosidase, ACE, DPPH) [15, 59]. Endophytic alkaloids also play a role in defense (herbivores, pathogens) of plants\ and can be associated with enhanced host alkaloid accumulation in medicinal species [64]. Endophytic fungi have shown the ability to biosynthesize alkaloids that are identical to those of their host plants. They have been found to produce bioactive compounds like paclitaxel, podophyllotoxin, vincristine, and camptothecin, previously considered unique to plants [60]. These results demonstrate their potential as alternative and sustainable sources of plant alkaloids. For example, specific endophytic fungi monitored in *Salvia abrotanoides*, including *Penicillium canescens* and *Paraphoma radicina*, can synthesize cryptotanshinone (a major bioactive compound from the host plant) independently of plant tissue. This phenomenon could be attributed to close metabolic alliances between endophytes and their host plants, where the fungal metabolic networks have the ability to utilize plant-derived precursors or imitate plant biosynthetic pathways [18]. Alkaloids include many compounds such as Aconitine, Berberine, Camptothecin, Huperzine A and others.

Aconitine, a diterpenoid alkaloid found in *Aconitum* spp. It is a highly toxic alkaloid, where is considered one of the most potent naturally occurring neurotoxins and cardiotoxins [86]. Moreover, aconitine is produced from endophytic fungus *Cladosporium cladosporioides* which isolated from *Aconitum leucostomum* plant [87].

Berberine is isoquinoline alkaloid compound that is found in several plants, including the roots, rhizomes, stems of some plants (such as *Berberis vulgaris*) as well as endophytic fungi [88]. The endophytic *Alternaria* sp. and *F.* solani, which were isolated from *Phellodendron amurense* and *Coscinium fenestratum* respectively were used for production of berberine [89]. Berberine is extensively utilized in the management of diseases such as hyperglycemia, hyperlipidemia and neural disorders. According to Singh, Sharma [90], berberine exhibited anticancer activity through binding to DNA and inhibiting replication.

Camptothecin (CPT) is an inhibitor of quinoline alkaloid that specifically targets topoisomerase I, resulting in cytotoxic effects. CPT is a naturally occurring compound derived from the bark and stem of *Camptotheca acuminata*. Its constituents have been utilized in traditional Chinese medicine for the treatment of cancer-related cases [91]. CPT and its derivatives have gained significant attention in the field of cancer research and chemotherapy. This substance is extremely effective as an inhibitor of topoisomerase I, an enzyme that plays a role in both the replication and repair of DNA. CPT causes DNA damage and ultimately cell death because it binds to the DNA-topoisomerase I complex which leads to the destruction of DNA [92]. CPT has been extracted from various fungal endophytes, includin *F. solani*,* Entrophospora infrequens*,* Neurospora sp* and *Nodulisiporium sp* [93–95].

Huperzine A is a natural compound derived from the Chinese club moss plant (*Huperzia serrata*). Also, many endophytic fungi such as *Aspergilli*,* Colletotrichum*,* Phyllosticta*,* Hypoxylon*,* Xylaria* and *Nigrospora* have been shown to synthesize novel strong acetylcholinesterase inhibitors (AChEIs) in their metabolite extracts [96]. Huperzine A works primarily as an acetylcholinesterase inhibitor. Acetylcholinesterase is an enzyme that breaks down the neurotransmitter acetylcholine, which is involved in memory, learning, and cognitive skills. By inhibiting this enzyme, huperzine A increases the levels of acetylcholine in the brain, which may help improve cognitive skills and memory [97].

### Terpenoids

Terpenoids are biosynthesized from C5 isoprene units through the mevalonate (MVA) or the methylerythritol phosphate (MEP) pathways. Endophytes are able to synthesize monoterpenoids, sesquiterpenoids, di-, sester-, and triterpenoids, as well as meroterpenoids that contain skeletons characteristic of both terpenoid and polyketide synthesis. Many novel terpenoids have been described from endophytic fungi within medicinal plants, crops, algae, mangroves, and marine systems [64]. Terpenes are classified to sesquiterpenes, diterpenes and meroterpenes. *Sesquiterpenes* are a category of terpenes characterized by their composition of 15 carbon atoms, which are obtained from the combination of three isoprene units. Xylarenones A and B, along with xylarenic acid, were extracted from *Xylaria* sp. NCY2 [98]. Silva et al. [99] reported the isolation of several cadinane sesquiterpene derivatives from *Phomopsis cassiae*, which was isolated from *Cassia spectabilis*. *Diterpenes* are a subclass of terpenes characterized by their composition of 20 carbon atoms, which are formed by the combination of four isoprene units. Paclitaxel (Taxol) is the most prevalent diterpene. Taxol, a diterpenoid with significant functional groups, is present in all species of the *Taxus* genus, although its first isolation occurred from *Taxus brevifolia*. There are many endophytic fungi have ability to produce Taxol such as *Fusarium solani*,* Alternaria sp.* and *Aspergillus sp.* which isolated *Taxus chinensis*,* Ginkgo biloba* and *Podocarpus sp*. respectively [100]. Furthermore, Zhao et al. [101] reported that various fungal species, including *Phyllosticta spinarum* and *Bartalinia robillardoides* have been identified as producers of Taxol. *Meroterpenes* are a class of natural compounds that are hybrid molecules containing both terpenoid and non-terpenoid moieties. They are formed through the combination of terpenoid precursor units with other biosynthetic building blocks, such as polyketides, shikimate pathway intermediates, or amino acids [102]. Fill et al. [103] reported that *Penicillium sp* has ability to produce meroterpenoids.

A lot of endophytic terpenoids display anticancer, anti-inflammatory, antibacterial, antiviral, antimalarial, antioxidant, and hypoglycemic activities [42, 64]. Terpenoids with antifungal and antibacterial activities are considered promising candidates for drug development and crop protection. However, their yields are typically low; therefore, optimization strategies, co-culture approaches, and omics-guided strain engineering represent key areas of research [64].

### Phenolics

Phenolics are aromatic compounds with one or more hydroxyl (–OH) groups, and in endophytes, they comprise simple phenols, phenolic acids, flavonoids, and tannins. Phenolic compounds are strongly correlated with antioxidative capacity, underscoring their role as the primary active constituents [104]. Extracts are investigated as natural antioxidants in foods (e.g., preservation of oxidation-sensitive olive oil) and lead to anti-cancer and anti-inflammatory compounds [105]. Fungal phenolics aid hosts in counteracting oxidative, biotic, and abiotic stress, strengthening the plant defense. Endophytes can also regulate plant phenolic pathways, leading to the accumulation of phenolic acid in medicinal plants [33]. Optimization of solvents, time, and culture conditions significantly enhances the recovery of phenolics [104]. Studies have shown that endophytic fungi could synthesize phenolics like their host plants. For instance, fungal isolates from *Lagenandra toxicaria* exhibited similar phytochemical profiles and antimicrobial properties as the plant itself [106]. The endophytes’ ability to produce phenolics like their host has been attributed to co-evolutionary processes and genetic communications enabling fungi to mimic or complement the metabolic pathways of the respective host [107, 108]. In a previous study, endophytic *Pestalotiopsis adusta* was used for production of pestalachloride A and B [109]. Also, Hoffman et al. [110] successfully extracted a collection of phenolic acids from the culture broth of a *Phoma* sp., which was identified as an endophytic fungus residing within a Guinea plant species known as *Saurauia scaberrinae*. Numerous novel phenols and phenolic acids, which have been extracted from endophytic cultures in recent research, have frequently exhibited significant antimicrobial properties [111]. Through conducting additional research on phenol and phenolic acids, there exists significant potential for the discovery of novel and highly efficacious antibiotics.

### Lignans and coumarins

Lignans and coumarins are bioactive phenolic compounds. Metabolomic analysis of endophytic fungal fermentation has shown that these compounds are among the main differential metabolites associated with therapeutic effects, exhibiting specific accumulation patterns during fermentation [112]. In *Cistanche sinensis*, a medicinal plant containing lignan compounds including syringin, the relationships between endophytic fungal communities and lignans indicated that endophytes might affect lignan biosynthesis or accumulation [113]. Endophytic fungi can produce lignans and coumarins that are identical or analogous to those of their host plants. This ability is attributed to the long-term co-evolution and genetic exchange between endophytic fungi and their host plants, which enables the fungi to biosynthesize secondary metabolites similar to plant-derived compounds [107, 108]. A notable example includes the production of podophyllotoxin, a lignan originally obtained from *Podophyllum* species, by endophytic fungi such as *Phialocephala fortinii*, highlighting the ability of endophytes to synthesize host-specific lignans [114].

Transcriptomic and metabolomic profiling of *Ganoderma lucidum* also reveal that lignans occur along with other phenolic compounds, suggesting possible fungal biosynthesis pathways, although the precise mechanisms are not yet completely understood. These compounds are responsible for antioxidant, anti-inflammatory, and antimicrobial properties reported for phenolic metabolites of endophytes, showing a consonance with their ecological functions and biotechnological profile [115].

### Polysaccharides

Polysaccharides derived from endophytic fungi have demonstrated important bioactivities, including antioxidant and antimicrobial effects, as evidenced by exopolysaccharides (EPS) from *Aspergillus fumigatus* and *Preussia isabellae*, which exhibit strong radical scavenging potential and antibacterial activity against Gram-positive bacteria [116]. Intracellular polysaccharides of endophytic *Penicillium* show strong antioxidant activity and can cause apoptosis in prostate cancer cells, without affecting normal cells [117]. The polysaccharide yield and production of endophytes can be improved through the optimization of culture conditions, as exemplified by *Acremonium* sp., where immobilization increased the intracellular polysaccharide content in addition to other bioactive compounds [118]. The anti-inflammatory activity of fungal polysaccharides is associated with structural characteristics, including glycosidic linkage of the carbohydrate moieties, in which different linkages modulate the gut microbiome and inflammation in colitis models [119]. Generally, fungal polysaccharides are interesting for pharmaceutical and nutraceutical usages because of their wide physiological activities and the possibility to upscale their production [120]. Endophytic fungi produce many polysaccharides with various structures and bioactivities. Studies have shown that some of these polysaccharides even mimic those found in their host plants [117]. A *Fusarium solani* endophyte has been reported to produce polysaccharides like those of its host plant *Dendrobium officinale* with the same monosaccharide composition and similar structure to the host Dendrobium polysaccharide (DOP), optimized via culture C/N ratio [121].

### Other bioactive compounds

Beyond alkaloids, terpenoids, phenolics, lignans, coumarins, and polysaccharides, endophytic fungi exhibit remarkable metabolic diversity, producing a wide range of bioactive compounds such as peptides, polyketides, steroids, and sulfur-containing molecules. These metabolites display a wide range of bioactivities, including antioxidant, anti-inflammatory, antimicrobial, anticancer, antiviral, and hemoprotective activities [122, 123]. New antibacterial compounds with distinct chemical structures are derived from endophytic fungi and have been considered as promising agents against antibiotic-resistant pathogens [122]. Certain secondary metabolites, such as averufin and equisetin, display effective anti-cancer activity by acting through molecular docking with cancerous proteins [124]. Fermentation of agricultural residues by endophytic fungi might be a potential approach to increase complex mixtures of amino acids, phenolics, flavonoids, tannins, polysaccharides, and proteins with multiple biological activities [125]. In general, endophytic fungi have a wide range of structurally diverse bioactive compounds that could serve as an enormous and largely untapped reservoir for pharmaceutically active compounds with good potential for therapeutic use [123].

## Enhancement of bioactive compounds production from endophytic fungi

Bioactive compound production in endophytic fungi is known to be an impending biotechnological frontier owing to a global demand for new pharmaceuticals, agrochemicals, and cosmeceuticals during the era of antibiotic resistance and increasing complex diseases. Endophytic fungi living asymptomatically in plant tissues are prolific producers of secondary metabolites with diverse structures and anticancer, antimicrobial, anti-inflammatory, and neuroprotective activities [126, 127]. Due to the silencing of biosynthetic gene clusters (BGCs) under standard culture conditions, their natural productivity in laboratories is normally low or inconsistent [128, 129]. Using several strategies, endophytic fungi can be stimulated to produce increased levels or novel secondary metabolites. Systematic variation of carbon/nitrogen sources, salts, pH, temperature, aeration, agitation, and incubation time (often employing statistical designs such as RSM) yields higher levels of certain metabolites as well as overall bioactivity [59, 130]. One Strain–Many Compounds (OSMAC) strategy, which involves media change, solid or liquid culture, and physical condition, awakens silent or weakly expressed biosynthetic gene clusters resulting in the production of various compounds [60, 131, 132]. Culturing endophytes with other fungi or bacteria simulates natural interactions, places stress on the fungi, and activates pathways that would otherwise go unexplored. Co‑culture of paclitaxel‑producing *Paraconiothyrium* sp. with the other endophytes increased paclitaxel up to 7.8‑fold [131]. Co‑culture of tropical endophytes reduced antibacterial IC50 ~ 10‑fold compared with monocultures [133]. Fungi co-cultivated with host plants or medicinal species can also stimulate plant metabolite accumulation [118]. The addition of elicitors (stress signals, small molecules) or precursors enhances target metabolite pathways and yields [15]. Emission of plant extracts or host‑derived material can augment production; *Huperzia serrata* extracts, for example, increased huperzine A yield in an endophyte to 255 µg/g dcw and induced biosynthetic genes [134]. Chemical epigenetic modifiers, including 5-azacytidine (AZA) and suberoylanilide hydroxamic acid (SAHA), along with DNA methyltransferase and histone deacetylase inhibitors, can remodel chromatin structure, activate silent biosynthetic gene clusters, and enhance secondary metabolite diversity [132, 135]. Manipulation of BGCs, global regulators, transcription factors, heterologous expression of clusters in model hosts, and CRISPR‑Cas9 editing are used to upregulate key genes or eliminate repressors [136]. Submerged culture is quicker and more easily managed; solid-state fermentation can lend preference towards different metabolic profiles. Process parameters such as aeration, agitation, inoculum size, and particle additives are tuned to maximize specific metabolite yields [59]. Classical or modern mutagenesis, protoplast fusion, and adaptive evolution are able to enhance the stability and productivity of endophytic strains [137]. Furthermore, Enhancement of bioactive metabolite production from endophytic fungi can be effectively achieved through gamma irradiation, which induces genetic variations that lead to increased yield and improved antimicrobial, anticancer, antioxidant, and other pharmacologically active compounds [63, 138].

## Biomedical applications of endophytic fungi

**Fig. 4:**
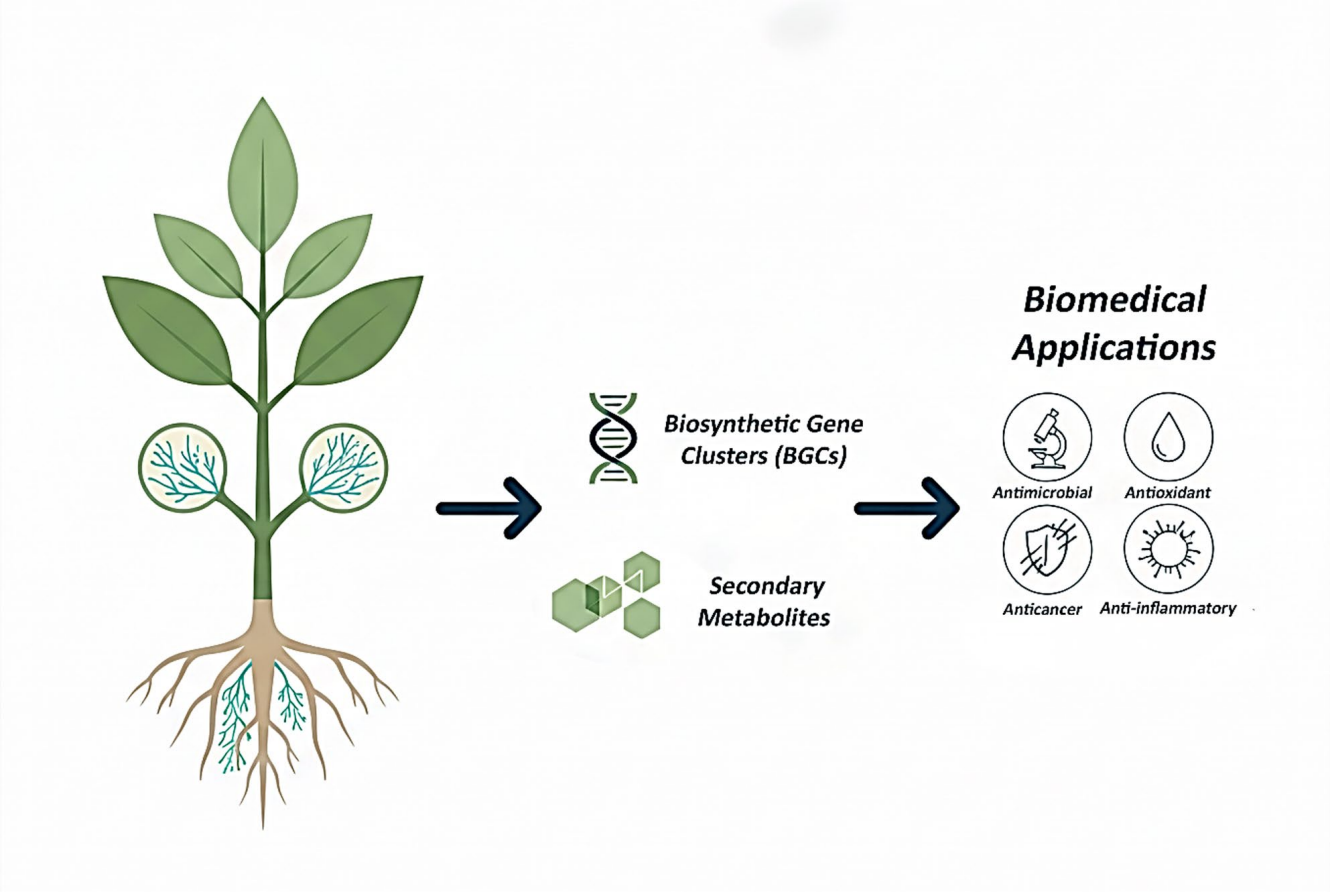
Endophytic fungi–derived metabolites: production and biomedical applications

Endophytes of medicinal plants are known to be important reservoirs of bioactive compounds, which have been demonstrated to possess various biological activities such as antimicrobial, anticancer, antioxidant, anti-inflammatory, and antidiabetic (Fig. 4). These fungi mainly produce a range of secondary metabolites such as taxol, camptothecin, podophyllotoxin, and several other new compounds, which can replace synthetic drugs and are effective against multidrug-resistant organisms [14, 60, 139].

### Antimicrobial activity

**Fig. 5:**
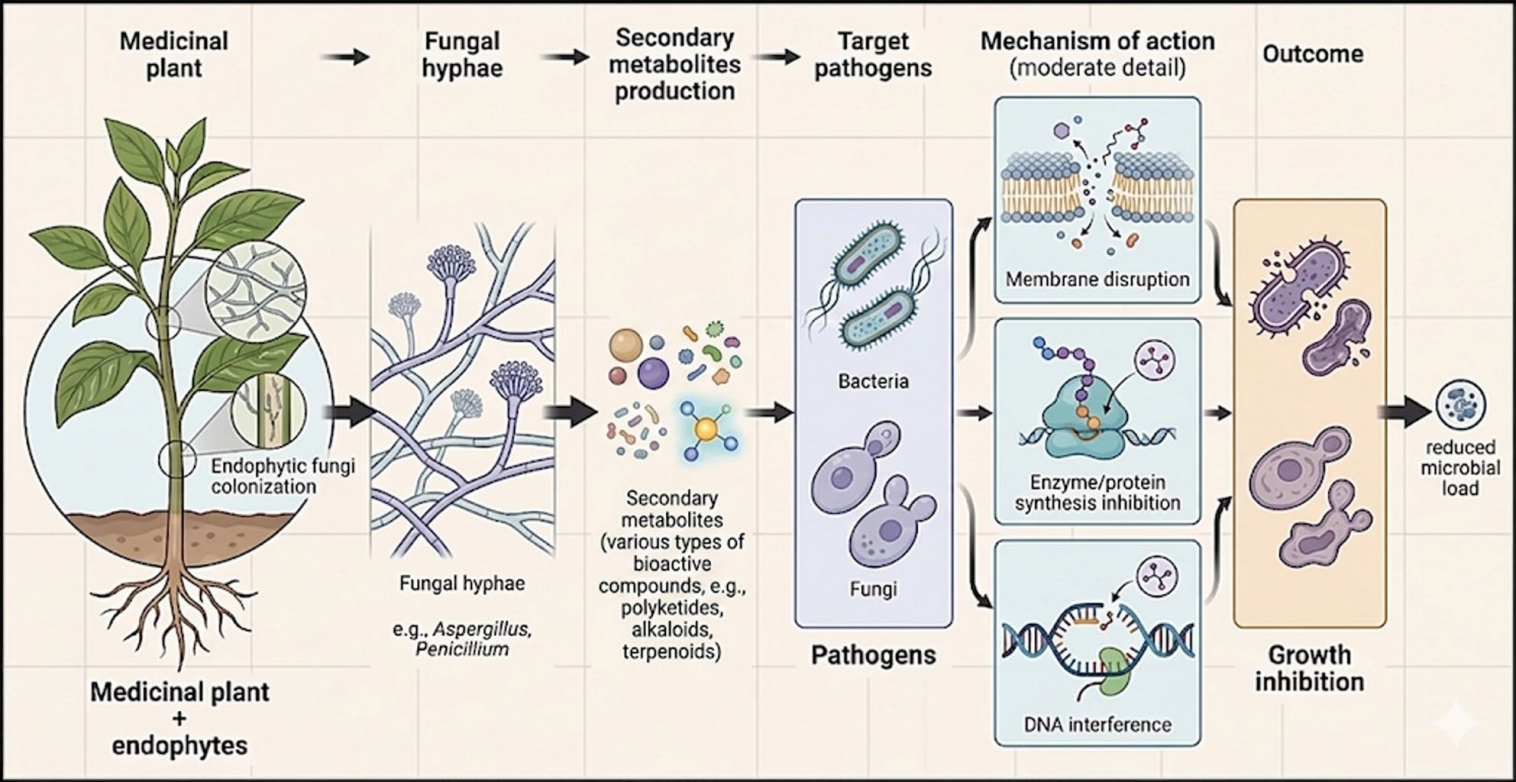
Mechanism of action of antimicrobial activity of endophytic fungi

The endophytic fungi obtained from medicinal plants revealed remarkable antimicrobial potential against a wide variety of human pathogens, such as bacteria and fungi. For example, *Aspergillus terreus* isolated from Egyptian medicinal plants, including *Anabasis setifera*,* Mesembryanthemum forsskaollii*,* Raphanus raphanistrum*,* and Zygophyllum coccineum*, has demonstrated significant antimicrobial activity against several human pathogenic bacteria, yeasts, and fungi, as well as antioxidant and cytotoxic activities [30, 140]. Likewise, antibacterial activity of South African and Amazonian medicinal plants endophytic fungi with minimum inhibitory concentration (MIC) in the low mg/mL range is associated with phenolic compound-related antioxidant potential [104, 141]. Endophytic fungi such as *Fusarium incarnatum* and *Fusarium oxysporum* from *Jatropha multifida* L. roots generated antibacterial compounds active against *Staphylococcus aureus* and methicillin-resistant *S. aureus* (MRSA) [142]. *Penicillium citrinum* and *Aspergillus* spp, was found by other researchers from *Garcinia cowa* and *Rhodomyrtus tomentosa*, respectively. which showed significant anti-bacterial activities against *S. aureus*,* E. coli*, and MRSA [143, 144]. Among the antimicrobial secondary metabolites produced by these fungi are cadinanes and abietane terpenoids, diterpenes, and depsides with antibiofilm activity, such as penicillic acid and methyl chloroacetate, suggesting their possible use as new natural antibiotics against resistant infections [145, 146]. These endophytes generate bioactive metabolites by multiple molecular mechanisms with antimicrobial properties (Fig. 5). These metabolites are believed to induce microbial cell membrane permeability by directly disrupting membrane integrity, causing loss of membrane potential, or indirectly leading to cell death [141]. For example, penicillic acid isolated from an endophyte of *Hyssopus officinalis* was shown to have strong antibacterial activity against both Gram-positive and Gram- negative bacteria in addition to reducing biofilm formation, suggesting that it targets bacterial growth as well as biofilm development [145]. Various compounds, including 2,2,4,4-tetramethylpentane, from *A. niger* (isolated from *Calotropis procera*) have been mechanistically linked to antimicrobial activity by forming stable adducts with bacterial enzymes (e.g., the phenylalanine-tRNA ligase and DNA gyrase) and inhibiting protein synthesis and DNA replication [147]. Various metabolites (-)-phomalactone demonstrated strong antibacterial activity against MRSA through enzyme inhibition and membrane disruption from *Curvularia inaequalis* [148]. Moreover, extracellular enzymes derived from endophytic fungi (e.g., proteases, laccases, and peroxidases) also exhibit antibacterial activity by degrading microbial components or disrupting quorum sensing and biofilm formation [149]. Overall, endophytic fungi constitute a valuable source for identifying novel antimicrobial compounds derived from medicinal plants [14].

### Antiviral activity

Endophytic fungi residing in medicinal plants constitute a valuable reservoir of diverse bioactive compounds, many of which exhibit significant antiviral potential against specific viruses, such as HIV-1, influenza A, and coronaviruses. For example, *Penicillium rubens* derived from *Albizia adianthifolia* exhibited potent anti-HIV-1 activity, with metabolites such as diosgenin exhibiting very high binding affinity to HIV-1 protease, which suggests the possibility of designing a novel antiviral agent [150]. Moreover, *Alternaria alternata* extracts also had low inhibitory concentrations against HIV-1, and such compounds are already reported to have antimicrobial and antioxidant properties that could be repurposed as anti-HIV agents [151]. An endophyte-derived metabolite (APL-16-5) produced by *Aspergillus sp*. has been shown to inhibit influenza A virus by promoting degradation of a viral polymerase subunit, and to confer protection in mice against lethal infection [152]. An endophytic *A. flavus* culture also demonstrated anti-viral activity against human coronavirus HCOV 229E, and the molecular docking results confirmed recognition of metabolites of this strain by viral proteases [153]. Other fungal metabolites like hexadepsipeptides and 4-hydroxy-2-pyridones isolated from *Fusarium* sp. also exhibited in vitro antiviral activity against coronaviruses [154]. Fungi produce a wide array of secondary metabolites that hinder viral replication by inhibiting key viral enzymes such as proteases and polymerases, preventing viral maturation and proliferation [152, 153]. Inhibition of viral protease activity and subsequent disruption of virus assembly or release is a mechanism by which fungal metabolites exert their antivirulence [150]. Another mechanism is through the induction of viral protein degradation using the host’s ubiquitin-proteasome pathway, such as a metabolite from *Aspergillus* sp. This leads to the degradation of influenza A virus polymerase subunit PA, thereby blocking viral replication [152]. Furthermore, many of these metabolites are characterized by antioxidant and anti-inflammatory roles to modulate host immunity response and reduce virus-mediated cellular injury, thereby promoting antiviral effectiveness [64].

### Anticancer activity

**Fig. 6:**
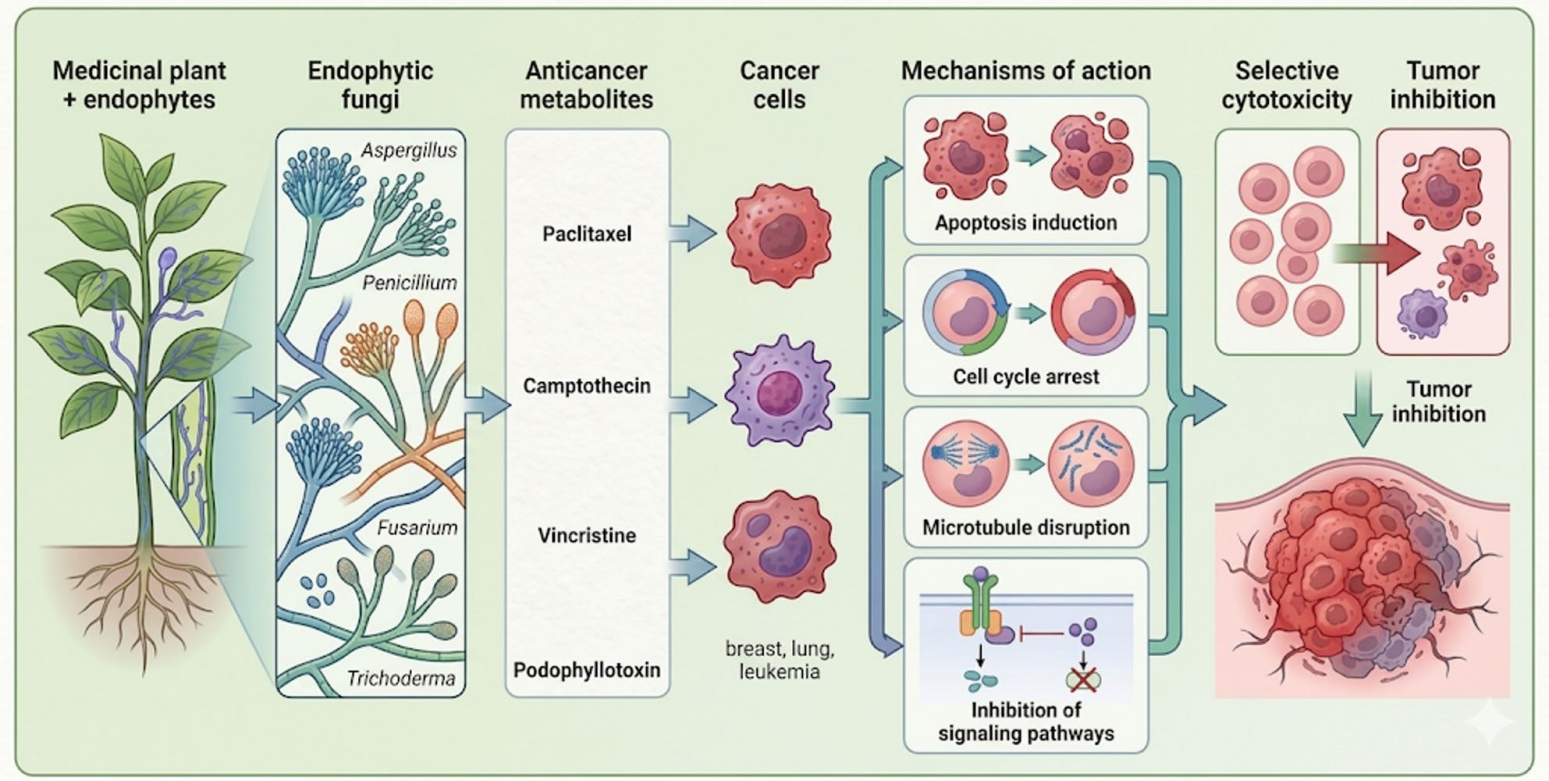
Mechanism of action of anticancer activity of endophytic fungi

Fungal endophytes are endosymbiotic microbes and have been considered as a valuable source of anticancer lead molecules for new drug discovery. These fungi have been shown to synthesize various bioactive metabolites like paclitaxel, camptothecin, vinblastine, vincristine, and podophyllotoxin, etc., which are already used in clinics against breast, lung, and ovarian carcinoma as well as for leukemia [14, 155]. Endophytic fungal extracts have demonstrated cytotoxic activity against various cancer cell lines, such as melanoma, breast adenocarcinoma, lung carcinoma, and hepatocellular carcinoma, showing selective toxicity toward malignant cells while sparing normal cells [156, 157]. Recent studies suggest that fungal endophytes can enhance the performance and anticancer efficacy of processes such as gamma irradiation and nanoparticle biosynthesis, thereby improving overall biological activity and biocompatibility [156, 158]. Various mechanisms are responsible for the anticancer activities of bioactive metabolites from endophytic fungi isolated from medicinal plants (Fig. 6); however, cytotoxicity plays a key role since cancer cells are killed in preference to normal cells. These metabolites include a wide range of chemical classes, including terpenoids, phenolics, flavonoids, and alkaloids that have been shown to trigger apoptosis, suppress cell proliferation, and disturb signaling pathways in cancer cells. For example, averufin from *Penicillium* sp. has high binding rates to tubulin proteins, disrupting microtubule dynamics that are crucial for the division of cancer cells [124]. Other identified bioactive compounds, such as squalene and α-phellandrene, increase their cytotoxic effects on melanoma, breast adenocarcinoma, and lung carcinoma cells [156]. Similarly, increased levels of metabolites produced by endophytic fungi work in various positions by modulating oxidative stress and inflammation when coming into contact with normal cells or enhancing immune responses against cancerous cells that further build anticancer potential [125]. Although many studies are in vitro and need further in vivo confirmation, the increasing evidence indicates that endophytic fungi are valuable and promising sources of new anticancer agents through mechanisms such as apoptosis induction, cell cycle arrest, and targeting cancer-specific proteins [155, 159]. Certain types of fungi, such as *A. niger* and *Penicillium* spp., have been shown to produce anti-cancer drugs of strong activity that also act by suppressing tumor cell growth and mobility [147, 157]. Herein, endophytic fungi represent a promising and sustainable reservoir for discovering new anticancer drugs with potential applications in breast cancer and other malignancies [160, 161].

### Anti-inflammatory activity

Endophytic fungi derived from medicinal plants produce many secondary metabolites having a high potential of anti-inflammatory activity as alternatives to existing drugs. Compounds derived from fungi, such as Alternaria species, have been found to promote macrophage polarization toward an anti-inflammatory phenotype, with altertoxins identified as potent anti-inflammatory agents [162, 163]. Oxygenated isocoumarins and xanthones isolated from mangrove endophytes have demonstrated inhibitory effects on nitric oxide production as well as on key inflammatory enzymes, including inducible nitric oxide synthase (iNOS) and cyclooxygenase-2 (COX-2), underscoring their therapeutic potential [164, 165]. Other endophytes, such as *A. niger* and *Nigrospora zimmermanii*, also gave rise to metabolites that restrained potent pro-inflammatory mediators like prostaglandin E2 and accelerated wound healing by a combination of antimicrobial and tissue regenerating modes of action [166, 167]. Molecular docking simulation studies also corroborate the association of these fungal secondary metabolites with inflammatory targets (TNF-α, IL-1β, INF-γ) and provide rationale for their anti-inflammatory activities [162, 168]. These endophytes show anti-inflammatory activity through many mechanisms, such as inducible nitric oxide synthase (iNOS), cyclooxygenase-2 (COX-2), and pro-inflammatory cytokines such as TNF-α and IL-6. Nitric oxide (NO) production in lipopolysaccharide (LPS)-stimulated macrophage cells is suppressed by compounds such as benzofuran derivatives, sesquiterpenoids, oxygenated isocoumarins, xanthones, and diterpenoids [164, 165, 169]. Certain metabolites, such as ravenelin and physcion, act at the molecular level by inhibiting both iNOS and COX-2 expression to modulate inflammation [168]. Other bioactive compounds, such as azaphilones, were also identified that reduce the production of pro-inflammatory cytokines and have been shown to be able to induce an anti-inflammatory phenotype in immune cells [163, 170]. They tend to utilize mechanisms such as downregulating inflammatory enzyme expression or reacting towards oxidizing species, both of which can contribute to the generation of antioxidant effects, which are then potentiated to support anti-inflammatory activity [141, 168]. The results not only identified bioactive molecules from endophytic fungal metabolites but also provided a mechanistic basis for the development of natural anti-inflammatory drugs [165, 166].

### Antioxidant activity

The endophytic fungi hosted by medicinal plants have some powerful antioxidant properties due to the secretion of various bioactive metabolites, including phenols, flavonoids, and diterpene lactones. Fungal extracts, including *A. terreus* and *Pestalotiopsis neglecta*, have shown a high free radical scavenging activity when tested against DPPH and ferric reducing antioxidant power (FRAP) assays, and a few have even found to possess antioxidant power in much the same way as reference antioxidants quercetin [30, 104, 171]. Such fungal extracts also prevent oxidative stress-induced DNA damage, suggesting possible uses for the prevention of oxidant-related diseases [171]. Antioxidant capacity is commonly correlated with high total phenolic content, as well as the presence of compounds such as tolycaine, eugenol, and various phenolic acids identified by chromatographic techniques [139, 172]. These endophytes are known for major antioxidant activities mainly through radical scavenging and metal ion chelation mechanisms, and bioactive metabolites. These fungi produce phenolic compounds, flavonoids, and various secondary metabolites such as kojic acid and diterpene lactones that contribute to their antioxidant potential by scavenging free radicals, including DPPH, ABTS, hydroxyl, and superoxide anions [171–173]. The antioxidant effect is frequently associated with the total phenolic and flavonoid concentration in fungal extracts, suggesting these bioactive compounds are largely responsible for combating oxidative stress [173]. The protective effect of fungal metabolites on DNA and their FRAP role also supports their cellular protection mechanism [104, 141]. Through multiple analytical approaches, including GC-MS and LC-MS, diverse bioactive compounds with therapeutic potential, such as phenols, terpenes, and saponins have been identified from endophytic fungi, many of which display extensive antimycotic and cytotoxic properties [174]. The mechanisms mainly consist of scavenging reactive oxygen species (ROS), chelating metal ions that catalyze ROS formation, and protecting biomolecules from oxidative damage, so it’s thought that endophytic fungi are a potential source of novel antioxidants with pharmaceutical properties [139, 171]. Some endophytes manufacture the same or similar secondary metabolites as those in their host plants, which can be sustainable sources of antioxidants for pharmaceutical purposes [175].

### Antidiabetic activity

Recent findings provide strong evidence supporting the antidiabetic potential of fungal endophytes from medicinal plants, and they exhibited mechanisms, such as enzyme inhibition and metabolic modulation. A few fungi, such as *Schizophyllum commune*, isolated from *Aloe vera*, showed in vitro α-glucosidase inhibition and reduced blood glucose levels, with beneficial effects on lipid profiles and antioxidant status in diabetic rats [176]. Methanolic extract of *Aspergillus austwickii* showed promising activities against α- and β-amylase enzymes, which are considered plausible targets for the management of postprandial hyperglycemia [177]. New diketopiperazine alkaloids from mangrove endophytic *Aspergillus* sp. exhibited potent inhibitory activity against α-glucosidase and are suggested for drug development [178]. In a related study, exopolysaccharides of *P. janthinellum* were shown to decrease blood glucose and increase insulin sensitivity in diabetic mice, demonstrating a wide range of bioactive compounds being produced by endophytes [179]. A few in silico studies have reported that a few fungal metabolites could inhibit targets such as α-amylase and sodium-glucose cotransporter 2 (SGLT2); further experimental evidence is required for their antidiabetic potential [180–182]. One of the most popular mechanisms of antidiabetic activity of bioactive compounds produced by endophytic fungi is the inhibition of carbohydrate-digesting enzymes in the small intestine, specifically α-glucosidase and α-amylase, which takes place by competitively inhibiting these brush-border enzymes. Fungal metabolites, such as phenolic compounds, delay the breakdown of complex carbohydrates into absorbable monosaccharides like glucose [183]. In addition to modulating glucose uptake into the blood, a number of fungal metabolites directly augment insulin sensitivity in end organs such as skeletal muscle and adipose tissue. This is done by activating intracellular signaling pathways. The AMP-activated protein kinase (AMPK) pathway is a central player in this process. When activated by increased cellular AMP/ATP ratios, a condition mimicked by the presence of particular metabolites, AMPK serves as a master metabolic regulator needed to stimulate ATP production and limit excessive energy consumption. It enhances glucose uptake by driving translocation of the glucose transporter, GLUT4, to the plasma membrane independent of insulin. This mechanism is similar to the main action of the most prescribed diabetes medicine, metformin, which also operates on AMPK activation [184]. Most importantly, endophytic fungi is an excellent resource of natural products to introduce new antidiabetic agents with multiple activities [14, 60].

### Antiparasitic and antimalarial activity

Endophytes from medicinal plants have enormous potential for the discovery of new antiparasitic and antimalarial drugs based on their ability to synthesize several diverse bioactive metabolites. These fungi provide a sustainable source of new compounds, which may be of therapeutic value in combating drug resistance in parasitic diseases such as malaria, by providing promising lead compounds for pharmaceutical research [132]. Endophytes produce diverse bioactive secondary metabolites such as alkaloids, flavonoids, terpenoids, quinones, peptides, and polyketides that exhibit antiparasitic and antimalarial activities by targeting key parasite enzymes or pathways [185]. Notable examples include erucamide, produced by *A. flavus* isolated from red ginger, with significant inhibition of *Plasmodium berghei* growth, presumably due to its binding to Plasmepsin I, which is an important enzyme in the malaria parasite [186]. Furthermore, it was shown through metabolomics and in silico prediction that quinone derivatives, including emodin and physcion from fungal endophytes of *Artemisia annua*, possessed significant antimalarial activity (IC50 values in the low micromolar range) [187]. Novel cyclic peptides such as pipecolisporin isolated from *Nigrospora oryzae* demonstrate antimalarial and antitrypanosome activity without notable cytotoxicity, supporting selective intervention in parasites [188]. Mechanisms include interference with proteases involved in parasite metabolism, as well as targeting metabolic enzymes and disrupting cellular processes critical for parasite survival or replication [189]. Despite the limited research on the antiparasitic potential of fungal endophytes, the broad-spectrum antimicrobial and bioactive properties of their metabolites indicate promising prospects for their application in the treatment of parasitic infections. Genomics and metabolomics are expediting the discovery and optimization of these fungal compounds for therapeutic purposes [132]. In general, endophytic fungi are an unexplored and promising source to obtain new antiparasitic and antimalarial drugs from medicinal plants [132, 190, 191].

## Pharmaceutical drug development

**Fig. 7:**
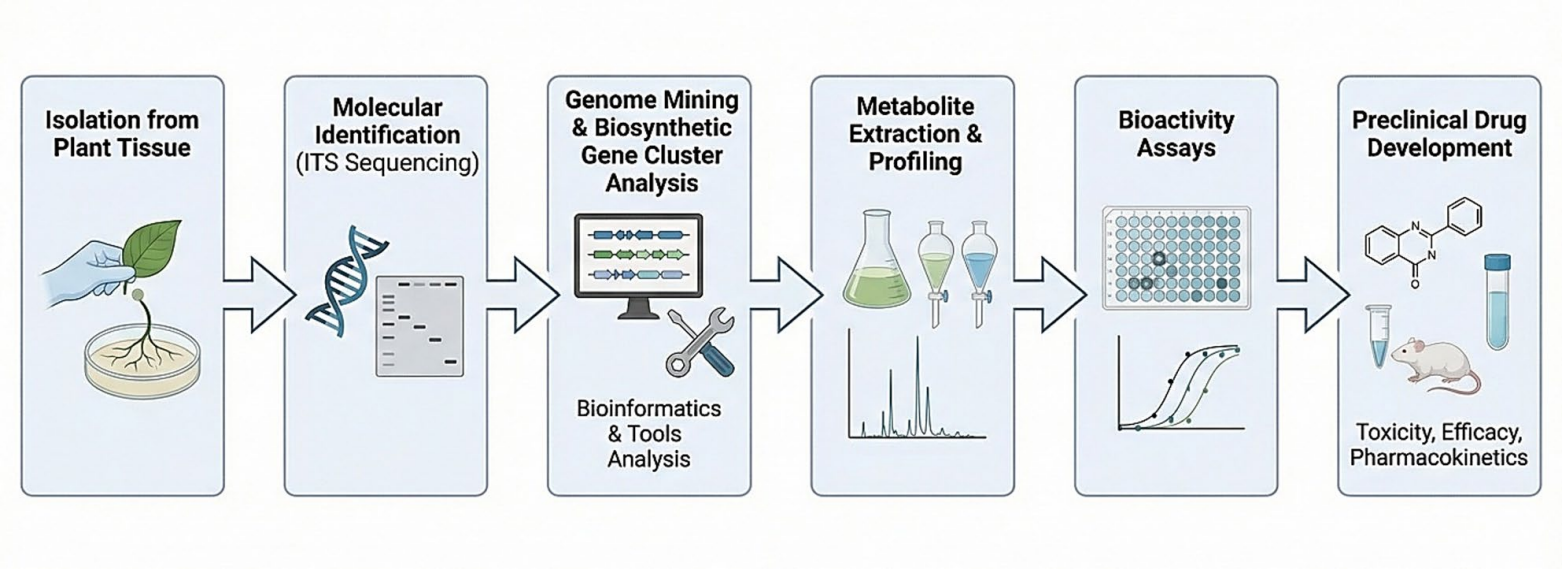
Drug development from endophytic fungi

Endophytic fungi from medicinal plants are sources of bioactive secondary metabolites that have great potential in the development of pharmaceutical-grade products that could be beneficial with antibacterial, antimicrobial, and enzyme-producing activities. Drug development from endophytic fungi includes fungi isolation from plant tissues and molecular identification using Internal Transcribed Spacer (ITS) sequencing. It then progresses through genome mining, metabolite profiling, and bioactivity screening, ultimately leading to preclinical drug development involving toxicity, efficacy, and pharmacokinetics evaluation (Fig. 7). Endophytic extracts isolated from *Psychotria poeppigiana* contain terpenes, flavonoids, and phenolic compounds with strong antibacterial activity against Gram-positive and Gram-negative bacteria and may be of use in formulating new antimicrobial drugs [192]. Fungal enzymes like L-glutaminase from *A. tamarii* possess potential use as therapeutic agents for their antimicrobial activity and stability under extreme conditions, with strong applicability in pharmaceutical biotechnology [193]. It can be applied as a therapeutic agent to inhibit pathogenic microorganisms and as a potential anticancer enzyme by depleting glutamine required for tumor cell proliferation. Additionally, its robustness supports its use in stable drug formulations and biocatalytic processes for pharmaceutical synthesis [194]. Further insight into the regulation of fungal secondary metabolism by histone post-translational modifications might allow increased production of useful compounds for drug applications [195]. Moreover, the combination of ethnic knowledge on natural drugs from plants with advanced quality control techniques can guarantee the safety and effectiveness of fungal-based drugs [196]. Thus, endophytic fungi are an abundant source for potentially important new drug leads against infectious diseases and other medical issues [192, 193, 195, 196].

## Genes associated with secondary metabolite biosynthesis

Omics-driven research reveals that the medicinal plant endophytic fungi are enriched with diverse BGCs for nonribosomal peptide synthases (NRPS), polyketide synthases, and PKS–NRPS hybrids, responsible for the production of a plethora of bioactive molecules. They are discovered, activated, and functionally connected with specific secondary metabolites by means of genome mining, transcriptomics, and epigenetic engineering tools. Integration of HyViS enables dozens of BGCs to be identified in individual endophytic strains using whole-genome sequencing and antiSMASH [197]. Toxicity, virulence, stress, and plant interaction are controlled by NRPS/PKS products. In entomopathogenic plant symbiotic fungi, such as *Beauveria*, PKSs and NRPSs gene clusters are predicted for tenellin, beauvericin, oosporein, bassianolide, and others contributing to virulence, cell wall integrity, UV or oxidative stress tolerance, and immune invasion [198–200]. Phytotoxic polyketides in *Alternaria* spp. are produced by PKS and PKS-NRPS, where the pair of reducing and non-reducing PKS genes (ALPK7/ALPK8) is involved in benzenediol lactones biosynthesis [201]. Transcriptomics has further shown that a *B.bezdeki* PKS15 orthologue is required for the expression of 36/45 remaining SM clusters (a number being NRPS/PKS) and suggests cross-pathway regulation by multiple toxins and siderophores [199]. Deletion of a PKS–NRPS gene (BBA_09856) in *B. bassiana* affects the conidial blastospore and enhances virulence and mycoparasitism to *Botrytis* [200]. In endophytes, most NRPS/PKS clusters are cryptic under normal growth conditions. Epigenetic modification (histone deacetylase/DNA methyltransferase inhibitors) modifies the chromatin and can lead to selective up- or down-regulation of the NRPS/PKS clusters, resulting in new chemical profiles [83, 135]. Reviews have featured genome mining combined with epigenetic elicitors and CRISPR-based editing to switch on, refactoring, or over-expressing NRPS/PKS BGCs in endophytic fungi for drug discovery [83, 135, 202].

## Application of genomics, transcriptomics, and metabolomics in endophyte research

**Fig. 8:**
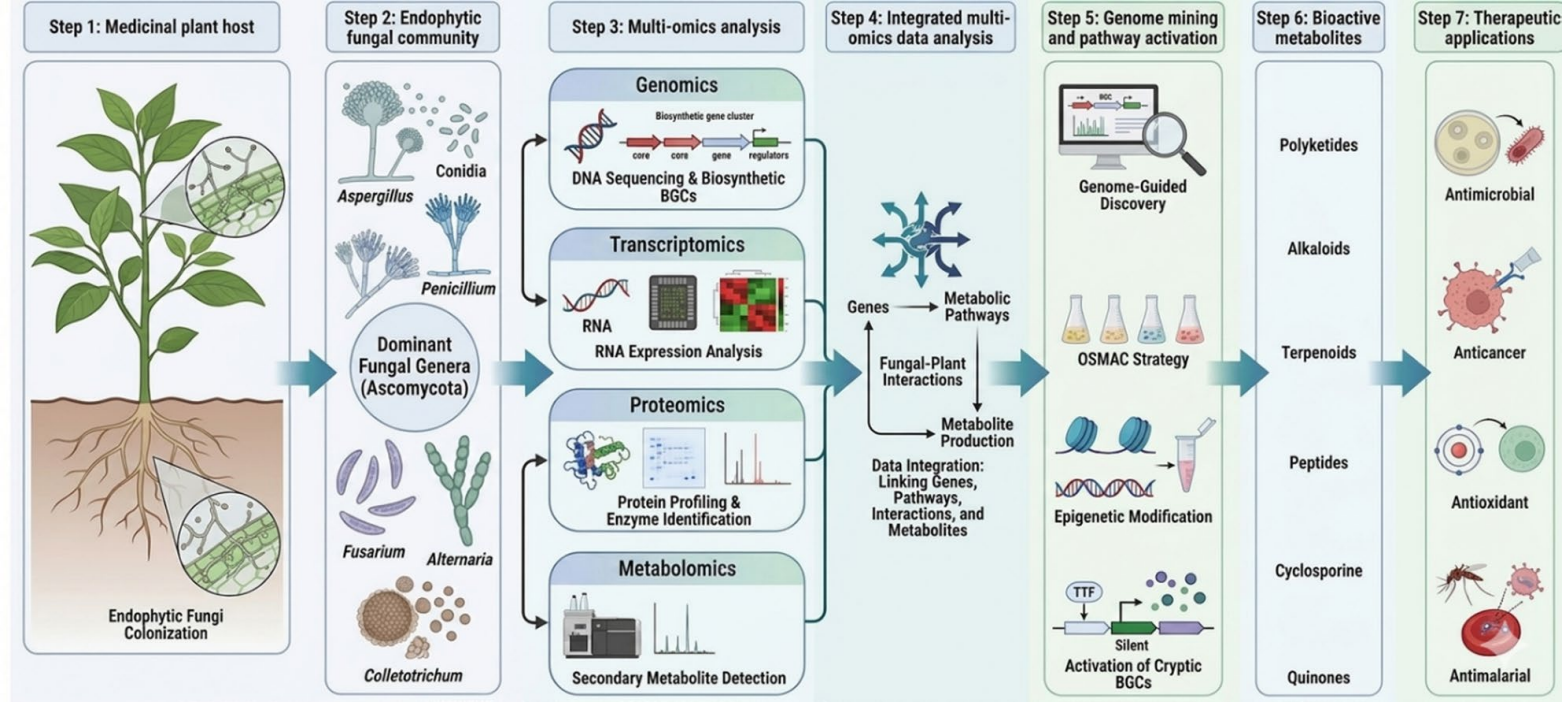
Workflow for discovering bioactive compounds from endophytic fungi of medicinal plants via multi-omics approaches

Genomics, transcriptomics, and metabolomics collectively provide a systems-level framework for understanding the biosynthetic potential and functional activity of endophytic fungi, enabling the rational discovery and enhancement of bioactive metabolites (Table 3; Fig. 8). The multi-omics approach facilitates establishing links among genes, pathways in fungus and host with real bioactive metabolites, and plant traits in terms of stress tolerance and disease resistance. Multi-omics indicates complex fungal communities (*Aspergillus*,* Penicillium*,* Fusarium*,* Alternaria* and *Colletotrichum*) in different medicinal plants, typically associated with the host and environmental conditions [2, 30, 203]. Genomic and multi-omics investigations have linked endophytic genes and BGCs to plant growth promotion, stress responses, and pathogen suppression through secondary metabolite production, as well as membrane signaling alterations [204, 205]. Whole-genome sequencing analysis of individual endophytes, such as *Fusarium* sp. VM 40, *Alternaria alstroemeriae* S6, and *Helotiales* sp. BL73 enables the decoding of BGCs encoding polyketides, NRPS-related products, terpenes, and Ribosomally synthesized and Post-translationally modified Peptides (RiPPs), highlighting their potential to biosynthesize biologically active compounds [206–208]. Genomic comparisons and genome mining (antiSMASH, CAZyme, KEGG, COG annotation) connect BGCs with putative metabolic pathways and ecological roles (e.g., plant association, stress tolerance) [135, 208, 209]. From the transcriptomics perspective, next-generation RNA-seq is also employed to map the expression of BGCs and plant-like pathways in endophytes, particularly those that produce metabolites identical or similar to host phytochemicals [209]. Global genome–transcriptome analyses are useful to distinguish active from inactive clusters and reveal which genes are induced during plant colonization or distinct culture conditions [64, 135, 209]. While at the metabolite validation stage, untargeted molecular networking by LC–MS/MS identifies the true metabolic products of endophytes, leading to the discovery of polyketide-lactones, quinones, alkaloids, terpenoids, and organic acids from medicinal-plant isolates [133, 207]. Metabolomics verifies genomic predictions (linking certain clusters to structures) and identifies metabolites up-regulated following epigenetic induction of cryptic BGCs [135, 206]. Metabolomics-mediated genome-guided mining in endophytes from medicinal plants (i.e., Vinca, Artemisia, Rosa Otoba, Veronica Anisomeles) revealed polyketides, alkaloids, terpenoids, peptides, quinones, phthalates, succinic acid, with antimicrobial, antitumor, antioxidative, and antimalarial properties [34, 187]. Epigenetic modification and OSMAC approaches, guided by genomics and transcriptomics, are applied to trigger cryptic BGCs and broaden chemical space [135, 210].

**Table 3:** Comparative overview of multi-omics approaches in endophytic fungi research

Omics approach	Core research question	Representative outputs	Refs.
Amplicon-based metagenomics	Which taxa are present, and how is the community structured?	ASVs/OTUs, diversity metrics (alpha/beta), identification of dominant and core taxa	[203]
Whole-genome sequencing	What biosynthetic and functional capabilities are encoded?	Biosynthetic gene clusters (BGCs), carbohydrate-active enzymes (CAZymes), virulence- and pathogenicity-related genes	[34]
Transcriptomics	Which genes are actively expressed during plant–fungus interaction?	Differentially expressed genes (DEGs) in host and endophyte, signaling pathways, and defense-related responses	[204]
Proteomics	Which proteins contribute to colonization and molecular communication?	Secreted proteins (secretome), cell wall–modifying enzymes, effector proteins	[64]
Metabolomics	Which metabolites are synthesized and accumulated in situ?	Molecular networking profiles, annotation, and characterization of secondary metabolites	[187]

## Recent studies integrating molecular and biochemical analyses

The recent research is concentrating more on integrating molecular identification and omics tools with traditional biochemical methods to comprehensively research endophytic fungi in medicinal plants as well as their metabolites. A recent study on endophytic fungi of medicinal plants demonstrates how a combination of metagenomics, transcriptomics, proteomics and metabolomics with biochemistry assays can follow interactions and growth promotion, but also the synthesis of secondary-metabolites from genes and transcripts via enzymes to the accumulation of metabolites [2]. A further review on endophyte–derived antimicrobial metabolites emphasizes the application of multi-omics (genomics, transcriptomics, metabolomics, proteomics) in combination with chromatography, MS, and NMR, as well as bioassays to circumvent low metabolite titres and stage-specific production, and to unravel host–endophyte communication pathways. Metabolomics-centred research focuses on linking LC-HRMS/NMR-based untargeted metabolomics with multivariate statistics, and in combination with, when available, genetic or culture manipulation approaches to prioritize strains and verify biological activities of metabolites of endophytic fungi. Genomics and metabolomics link biosynthetic gene clusters to secondary metabolites (extrolites) and their associated biological activities [207]. Metabolomics and classic isolation chemistry, correcting and refining automated MS-based annotations and identification of structures and activities [211]. Taken together, these combined molecular and biochemical techniques are moving endophyte research out of mere screening towards mechanism-resolved natural product discovery as well as host–microbe interaction analysis.

## From laboratory discovery to industrial application: yield improvement, scalability, and production challenges

Although endophytic fungi are increasingly recognized as promising biofactories for pharmaceutically and agriculturally important metabolites, the transition from laboratory-scale discovery to industrial production remains a major bottleneck. In laboratory conditions, many endophytic fungi produce a wide range of bioactive secondary metabolites; however, the yields are often low, unstable, and highly dependent on specific cultivation parameters due to the cryptic nature of biosynthetic gene clusters and their complex regulatory networks [212]. To overcome this limitation, strategies such as the OSMAC approach, co-culture systems, and epigenetic modulation have been widely applied to enhance metabolite diversity and yield at the laboratory scale [213]. Nevertheless, these improvements are frequently not sustained under industrial fermentation conditions. A key challenge in industrial translation is process scalability in bioreactors. While shake-flask experiments allow controlled optimization of fungal growth and metabolite production, large-scale bioreactors introduce complex physicochemical constraints, including oxygen transfer limitations, shear stress, mixing heterogeneity, and pH fluctuations. These factors can significantly alter fungal morphology, which in turn directly affects secondary metabolite biosynthesis and overall productivity [214, 215]. Consequently, production titers achieved at laboratory scale often decline sharply during scale-up, highlighting the lack of robust and transferable fermentation protocols for endophytic fungi. Another major limitation is strain instability over successive generations. Endophytic fungi may undergo genetic drift, epigenetic reprogramming, or loss of biosynthetic gene cluster activity during repeated subculturing or prolonged fermentation. This instability leads to inconsistent metabolite profiles and unpredictable yields, which is a critical barrier for commercial development. Furthermore, silent gene clusters that can be artificially activated under laboratory conditions may become re-silenced under industrial stress environments, reducing long-term production reliability.

From a bioprocessing perspective, yield optimization remains a central challenge. Although metabolic engineering and genome mining have enabled the identification of high-value biosynthetic pathways, the conversion of this genetic potential into high-yield production systems is still limited. Factors such as precursor availability, metabolic flux imbalance, feedback inhibition, and competition between primary and secondary metabolism restrict maximal metabolite output. In addition, downstream processing of fungal metabolites is often complex and costly due to low concentrations, structural similarity of compounds, and co-production of unwanted by-products. Commercialization is further constrained by economic and regulatory challenges. High production costs, lack of standardized industrial fermentation platforms, and limited regulatory frameworks for genetically modified fungal strains slow down market translation. Moreover, scalability of optimized laboratory conditions is not always economically viable, particularly when compared with plant extraction or chemical synthesis for certain compounds. Furthermore, while endophytic fungi offer a sustainable and versatile platform for natural product discovery, their industrial application requires significant advances in strain engineering, bioprocess optimization, and metabolic control. Future progress will depend on developing genetically stable high-producing strains, improving fermentation system design, and integrating systems biology with industrial biotechnology to bridge the gap between laboratory discovery and commercial-scale production [212, 216].

## Challenges and future perspective

Despite remarkable progress in the study of endophytic fungi as reservoirs of bioactive secondary metabolites, several critical challenges still limit their full biotechnological exploitation. One of the most important constraints is the poor reproducibility of metabolite production. Secondary metabolite profiles are highly sensitive to minor variations in culture conditions, including nutrient composition, pH, temperature, and co-culture interactions. As a result, compounds reported in laboratory studies are often difficult to reproduce consistently across different laboratories or scale-up conditions, which undermines their reliability for industrial applications. Another major challenge is the gap between genomic potential and metabolic output. Although genome mining has revealed a large number of biosynthetic gene clusters (BGCs), a significant proportion of these clusters remain transcriptionally silent under standard laboratory conditions. The regulatory mechanisms controlling their activation are complex and still not fully understood, making it difficult to predict or control metabolite production in a rational manner. From an applied perspective, industrial scalability remains a major bottleneck. Many endophytic fungi that produce valuable metabolites under laboratory conditions exhibit slow growth rates, unstable productivity, or altered metabolic profiles when transferred to large-scale fermentation systems. Variations in oxygen transfer, shear stress, and nutrient gradients in bioreactors can significantly affect fungal physiology and suppress secondary metabolism, leading to reduced yields. In addition, genome editing and synthetic biology applications face several technical limitations. These include low transformation efficiency, limited availability of species-specific genetic tools, unstable expression of introduced genes, and difficulties in achieving precise and stable editing using CRISPR/Cas systems. Furthermore, the complex regulatory networks governing secondary metabolism often lead to unpredictable outcomes after genetic manipulation, limiting the efficiency of pathway engineering strategies. Regulatory issues also represent a significant barrier, particularly regarding the biosafety and approval of genetically modified fungal strains. The absence of standardized global regulations for engineered endophytic fungi slows down their translation from laboratory research to commercial applications, especially in pharmaceutical and agricultural sectors.

Despite these challenges, the future of endophytic fungi research is highly promising, driven by advances in multi-omics technologies, synthetic biology, and systems-level metabolic engineering. The integration of genomics, transcriptomics, proteomics, and metabolomics will enable a more comprehensive understanding of biosynthetic pathways and regulatory networks, allowing for more accurate prediction and activation of silent gene clusters. Emerging approaches such as machine learning and AI-based genome mining are expected to play a key role in predicting metabolite structures and biosynthetic pathways, significantly accelerating natural product discovery. In parallel, the development of more efficient genome editing platforms, including improved CRISPR/Cas variants and species-adapted transformation systems, will enhance the precision and stability of metabolic engineering in non-model endophytic fungi. Furthermore, advances in heterologous expression systems and synthetic biology chassis organisms may provide reliable platforms for large-scale production of valuable fungal metabolites, overcoming limitations associated with native strains. Optimization of fermentation strategies, including bioreactor design and process control, will also improve scalability and industrial feasibility. Finally, the integration of eco-friendly and sustainable bioprocessing approaches will support the development of endophytic fungi as green biofactories for pharmaceuticals, agricultural agents, and industrial compounds. Herein, bridging the gap between omics-driven discovery and industrial application will define the next phase of innovation in this field.

## Conclusion

Endophytic fungi associated with medicinal plants have emerged as a promising and sustainable reservoir of bioactive compounds with significant biomedical applications. Their ability to produce diverse secondary metabolites, including alkaloids, terpenoids, phenolics, and polysaccharides, highlights their potential as alternative sources of valuable therapeutic agents. These fungi not only mimic host plant metabolites but also generate novel compounds with enhanced bioactivity, contributing to drug discovery and development. Advances in cultivation techniques and metabolic engineering strategies have improved the production of these compounds, although challenges such as low yield, instability, and silent biosynthetic gene clusters persist. The integration of multi-omics approaches, including genomics, transcriptomics, and metabolomics, has provided deeper insights into the biosynthetic pathways and regulatory mechanisms underlying metabolite production. These tools have facilitated the identification of key genes and pathways, enabling targeted manipulation for enhanced productivity. Furthermore, endophytic fungi play a vital ecological role in promoting plant growth, enhancing stress tolerance, and protecting against pathogens, making them valuable for sustainable agriculture. Despite existing limitations, ongoing developments in synthetic biology, genome editing, and fermentation technologies are expected to overcome current challenges. Future research should focus on large-scale production, standardization, and clinical validation of endophyte-derived compounds. In conclusion, endophytic fungi represent a highly promising, eco-friendly, and versatile platform for developing novel drugs and improving agricultural sustainability, with significant implications for human health and global food security.

## Data Availability

No datasets were generated or analysed during the current study.
